# Effect of Particle Sizes on the Efficiency of Fluorinated Nanodiamond Neutron Reflectors

**DOI:** 10.3390/nano11113067

**Published:** 2021-11-14

**Authors:** Aleksander Aleksenskii, Marcus Bleuel, Alexei Bosak, Alexandra Chumakova, Artur Dideikin, Marc Dubois, Ekaterina Korobkina, Egor Lychagin, Alexei Muzychka, Grigory Nekhaev, Valery Nesvizhevsky, Alexander Nezvanov, Ralf Schweins, Alexander Shvidchenko, Alexander Strelkov, Kylyshbek Turlybekuly, Alexander Vul’, Kirill Zhernenkov

**Affiliations:** 1 Laboratory of Physics for Cluster Structures, Ioffe Institute, Polytechnicheskaya Str. 26, 194021 St. Petersburg, Russia; blin@mail.ioffe.ru (A.A.); Dideikin@mail.ioffe.ru (A.D.); Avshvid@mail.ioffe.ru (A.S.); AlexanderVul@mail.ioffe.ru (A.V.); 2 National Institute of Standards and Technology Center for Neutron Research, Gaithersburg, MD 20899, USA; markusbleuel@gmail.com; 3 Department of Materials Science and Engineering, University of Maryland, College Park, MD 20742, USA; 4 European Synchrotron Radiation Facility, 71 av. des Martyrs, F-38042 Grenoble, France; alexei.bossak@esrf.fr (A.B.); aleksandra.chumakova@esrf.fr (A.C.); 5 Institut de Chimie de Clermont-Ferrand (ICCF UME 6296), Université Clermont Auvergne, CNRS, 24 av. Blaise Pascal, F-63178 Aubière, France; marc.dubois@uca.fr; 6 Department of Nuclear Engineering, North Carolina State University, Raleigh, NC 27695, USA; ekorobk@ncsu.edu; 7 Frank Laboratory of Neutron Physics, Joint Institute for Nuclear Research, 6 Joliot Curie, 141980 Dubna, Russia; lychag@nf.jinr.ru (E.L.); muz@nf.jinr.ru (A.M.); grigorijnekhaev@yandex.ru (G.N.); nezvanov@jinr.ru (A.N.); str@jinr.ru (A.S.); kilishbek-t@mail.ru (K.T.); k.zhernenkov@fz-juelich.de (K.Z.); 8 Faculty of Physics, Lomonosov Moscow State University, GSP-1, Leninskie Gory, 119991 Moscow, Russia; 9 Department of Nuclear Physics, Dubna State University, Universitetskaya 19, 141982 Dubna, Russia; 10 Institut Max von Laue–Paul Langevin, 71 av. des Martyrs, F-38042 Grenoble, France; schweins@ill.eu; 11 Faculty of Physics and Technology, L.N. Gumilyov Eurasian National University, Satpayev Str. 2, Nur-Sultan 010000, Kazakhstan; 12 The Institute of Nuclear Physics, Ministry of Energy of the Republic of Kazakhstan, Ibragimova Str. 1, Almaty 050032, Kazakhstan; 13 JCNS at Heinz Maier-Leibnitz Zentrum (MLZ), Forshungzentrum Julich GmbH, 1 Lichtenbergstrasse, G-85748 Garching, Germany

**Keywords:** detonation nanodiamonds, nanopowder, reflectors of slow neutrons, albedo, size separation of nanodiamonds, fluorination, Monte Carlo

## Abstract

Over a decade ago, it was confirmed that detonation nanodiamond (DND) powders reflect very cold neutrons (VCNs) diffusively at any incidence angle and that they reflect cold neutrons quasi-specularly at small incidence angles. In the present publication, we report the results of a study on the effect of particle sizes on the overall efficiency of neutron reflectors made of DNDs. To perform this study, we separated, by centrifugation, the fraction of finer DND nanoparticles (which are referred to as S-DNDs here) from a broad initial size distribution and experimentally and theoretically compared the performance of such a neutron reflector with that from deagglomerated fluorinated DNDs (DF-DNDs). Typical commercially available DNDs with the size of ~4.3 nm are close to the optimum for VCNs with a typical velocity of ~50 m/s, while smaller and larger DNDs are more efficient for faster and slower VCN velocities, respectively. Simulations show that, for a realistic reflector geometry, the replacement of DF-DNDs (a reflector with the best achieved performance) by S-DNDs (with smaller size DNDs) increases the neutron albedo in the velocity range above ~60 m/s. This increase in the albedo results in an increase in the density of faster VCNs in such a reflector cavity of up to ~25% as well as an increase in the upper boundary of the velocities of efficient VCN reflection.

## 1. Introduction

Slow neutrons are usually subdivided into three ranges: ultracold neutrons (UCNs) [1,2,3,4,5], very cold neutrons (VCNs), and cold neutrons (CNs). The characteristic feature of UCNs is their (nearly) total reflection from a material surface provided the neutron velocity is smaller than the critical velocity of the surface material; a typical value of the critical velocity of materials used to build UCN traps is ~5 m/s. The available UCN fluxes are extremely low; however, this property of total reflection makes them an invaluable tool in fundamental neutron physics. CNs are widely used in neutron scattering and particle neutron physics due to their much higher fluxes than those of UCNs. Their wavelengths are slightly larger than interatomic distances; thus, Bragg scattering in solids starts disappearing, and matter starts becoming more transparent. Most of the neutrons are thermalized in cryogenic CN sources [6] in research nuclear reactors and spallation neutron sources to this energy range. A typical velocity of CNs is ~500 m/s. The intermediate range of VCNs with a typical velocity of ~50 m/s is rarely used because of two reasons: they have much lower fluxes than those of CNs, and there is no storage in traps unlike in the case of UCNs. We explore the possibility of solving both of these problems by means of developing VCN reflectors, which are efficient in the lower half of the so-called “reflectivity gap”. 

Over a decade ago, it was confirmed that detonation nanodiamond (DND) powders reflect VCNs diffusively at any incidence angle, and they reflect CNs quasi-specularly at small incidence angles [7,8,9,10,11,12]. In analogy to UCNs, the phenomenon of efficient reflection of neutrons from DND powder could be used for the definition of the range of velocities/energies of VCNs. Potential applications of these phenomena in neutron technology, as well as the beauty and complexity of the phenomena themselves, motivate active experimental and theoretical research in this area [13,14,15,16,17,18,19,20]. 

Not only can neutrons be reflected from DNDs, but they can also be lost due to the interaction with DNDs in the course of diffusive motion. As neutron losses in raw DNDs are dominated by a small admixture of hydrogen on the surface of DNDs, the removal of hydrogen by fluorination [21,22,23,24,25] is essential for reducing neutron losses. 

The properties of DNDs have been studied over the decades of their research and use, in particular those relevant to neutron reflectors [26,27,28,29,30,31,32,33,34,35,36,37,38,39,40,41,42,43,44,45]. In relation to neutron reflectors, DNDs combine the large cross-section of coherent scattering and the low cross-section of neutron losses. As this paper shares the scientific motivations and some experimental methods with another article published in the same issue of this journal [46], we limit our description here to only the specific results related to the subject of our present research. We refer the reader to this previous publication for a detailed description of the principle of operation and the general properties of such neutron reflectors. In the present publication, we report results of our study to optimize the sizes of DNDs in order to increase the neutron albedo in the most interesting VCN range; the neutron albedo is neutron reflectivity for the isotropic angular distribution of incident neutrons. From below, the VCN velocity range starts directly from UCNs. The most probable VCN velocity at the only existing VCN user facility, PF2 at ILL, is ~50 m/s. The maximum available VCN velocity is ~200 m/s. Better VCN reflectors might allow for an increase in this value. 

For every neutron wavelength, there is an optimum nanoparticle diameter that corresponds to the maximum neutron transport cross-section. In the model of diamond nanospheres, the optimal particle diameter $D_{opt}$ is related to the neutron wavelength $\lambda_{n}$ and the neutron velocity $V_{n}$ as follows [8]:

(1) $$D_{opt}\left[\mathrm{nm}\right]\approx 0.54\lambda_{n}\left[\mathrm{nm}\right]\approx 215/V_{n}\left[m/s\right].$$

In particular, for a typical VCN velocity of ~50 m/s, the optimal particle diameter is ~4.3 nm, precisely in line with a typical size of commercially available DNDs. 

**Figure 1:**
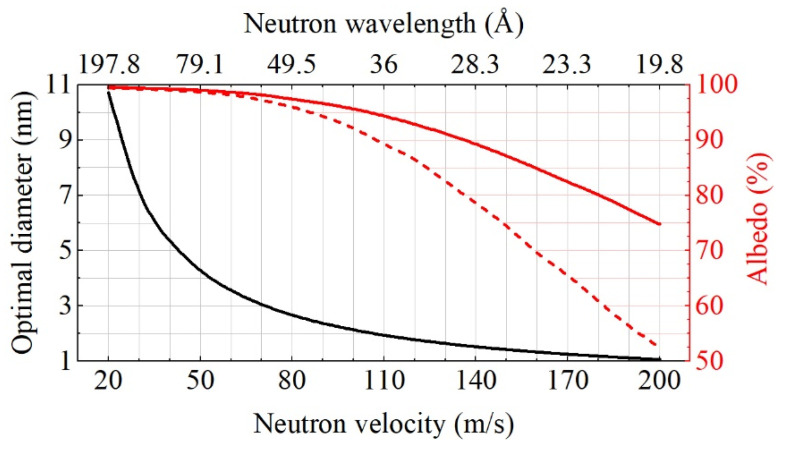
Optimal diameters of diamond nanospheres (black line) as a function of VCN velocity/wavelength. Solid red line shows the corresponding calculated VCN albedo for the optimal diameters of diamond nanospheres. Dashed red line stands for the VCN albedo from real DF-DNDs (see ref. [46]). Neutrons are only absorbed by carbon atoms.

Figure 1 illustrates the calculated optimal particle sizes and the corresponding maximum VCN albedo for a range of neutron velocities from 20 to 200 m/s.

This and all other calculations presented below were performed using Monte Carlo method and the model of discrete-sized diamond nanospheres (MDDNS) [17,46]. In the particular case shown in Figure 1, the powder layer thickness is 1 cm and the density is 0.56 g/cm^3^. All particles are assumed to have a spherical shape and monodisperse optimal diameter, at which the transport cross-section for each wavelength reaches its maximum. It is clear that for the broad range of velocities above ~50 m/s, the mean size of particles should be smaller than ~4.3 nm. For comparison with the ideal case, we added a dashed curve corresponding to the albedo calculated for a model of the real powder of deagglomerated fluorinated DNDs (DF-DNDs) with the same density of 0.56 g/cm^3^ described in ref. [46] (the sample with the best performance achieved). The difference between DF-DNDs and optimal diamond nanospheres is due to the deviation from the optimum size and the spread of sizes. 

Evidently, a maximum neutron albedo for all wavelengths at once is unattainable for any particular DND powder. However, the albedo can be increased in a broad wavelength range by properly selecting the mean size of DNDs. Therefore, we found a way to separate, by centrifugation, the fraction of finer DNDs from a broad size distribution in the initial powder and experimentally and theoretically studied the performance of a neutron reflector made of such particles. To underline the separated DNDs as well as the small particle size in this powder, we refer to these as S-DNDs to distinguish them from the DF-DNDs, which are used for comparison.

When choosing methods of characterization of DNDs, we took into account that our main goal was to describe the diffusion of neutrons in the DND powder. Therefore, SANS (small-angle neutron scattering) is the main characterization method used in this paper, as it allowed us to describe DNDs as neutron scatterers. Other methods are complementary and were used to confirm the effect of different procedures that we applied to modify the samples (fluorination, size separation, etc.). When applying MDDNS to SANS data, we approximated a real medium with a model one, the main goal of which was to precisely describe all of the SANS data and to extrapolate the experimental data to velocity and angle ranges that are not accessible for direct SANS measurements. Such a model allowed us to describe neutron diffusion in DND powders in all velocity and angular ranges of interest. 

This approach allowed us to take into account all relevant properties of the interaction of neutrons with nanostructured media described in this work (diamond cores and non-diamond carbons, nano-pores, interference on neighbor scatterers, etc.). The structures of significantly larger sizes, originating in particular from microporosity and microstructure, have no direct effect on neutron transport characteristics, as they result in the scattering of too-small angles. However, they are also included in our analysis through SANS measurements. The absence of neutron-absorbing impurities must be verified by other methods (neutron activation, neutron prompt-γ analysis, etc.) for each DND powder considered to be used for a real reflector.

There are several options for building neutron reflectors based on DND powders, and these include placing a DND powder in a thin-wall envelope made of materials with low neutron losses as used, for instance, for the first demonstration of VCN storage in a closed trap [10], as well as sintering and cold compaction, as is being investigated in the ANR-20-CE08-0034 project (ANR–Agence Nationale de la Recherche, France). The choice between these options depends on the particular applications of such reflectors. However, the results of the present study are valid for all these cases. 

The details of the sample preparation process are described in Section 2. In Section 3, we present the results of experimental studies of the particle size distribution. We investigated DF-DND and S-DND samples with complementary techniques as follows: Section 3.1 illustrates the size distribution of DNDs in these samples using transmission electron microscopy (TEM); in Section 3.2, the size distribution of DND cores using X-ray diffraction (XDR) is discussed; SANS results are described in Section 3.3; in Section 4, we discuss the calculated performance of DF-DND and S-DND reflectors and the effect of increased albedo on the yield of neutrons.

## 2. Samples

For compatibility with previous results [22,23,24,25,46], we used raw DNDs produced at the Federal State Unitary Enterprise, “Russian Federal Nuclear Center—Academician E.I. Zababakhin All-Russian Research Institute of Technical Physics” (FSUE “RFNC-VNIIF”), Snezhinsk, Russia, Technical Regulations TY 2-037-677-94. Their fluorination was carried out at the Institute of Chemistry of Clermont-Ferrand (Université Clermont Auvergne and CNRS), Aubière, France, in accordance with the procedure described in ref. [22], to obtain the fluorinated DND (F-DND) samples, which were then deagglomerated as described in ref. [46] to obtain the DF-DND samples.

While molecular fluorine does not react with sp^3^ carbons of the diamond core, it decomposes amorphous carbons when the reaction is carried out at a high temperature such as 450 °C in our case. The lower the crystalline order, the higher reactivity of sp^2^-type carbon. At this temperature, graphitized samples form, in pure F_2_ gas, a mixture of (C_2_F)*_n_* and (CF)*_n_* structural types, whereas amorphous carbons are decomposed into C*_x_*F*_y_* gases (*x* = 1, 2, 3, … and *y* = 4, 5, 6, …). As a gas/solid reaction, the F_2_ molecules react with the available sp^2^ C and functional groups (mainly C–OH, C–H, C=O, and COOH) on the diamond surface. If those groups are located on sp^2^ C shells, they are removed together with C*_x_*F*_y_* gases. When located onto the diamond surface, they are converted into C–F bonds. It is important to note that no diffusion of fluorine occurs inside the diamond core. At boundaries, two opposite cases may occur: F_2_ molecules open channels for their diffusion through the decomposition of sp^2^ carbons. If this phenomenon does not occur, the impurities located at boundaries are inaccessible for molecular fluorine, and their removal fails. 

We separated an S-DND fraction of finer DNDs from a broad size distribution. For this purpose, we used raw DNDs produced at the Federal State Unitary Enterprise Special Design-Technology Bureau (FSUE SDTB) “Technolog”, Saint-Petersburg, Russia. These DNDs underwent the deagglomeration process described in ref. [36]. The obtained hydrosol of deagglomerated particles was centrifuged (in a Sigma 6–16 centrifuge (SIGMA Laborzentrifugen GmbH, Germany) with the acceleration $a_{max}=1.8\times 10^{4}\;g$ for 100 min) in order to separate particles by size in water. The supernatant containing S-DND particles with diameters of ~3 nm was carefully separated from the sediment. Note that such diameters are well within the range of stability of DNDs, whose lower bound is ~1.2 nm [33].

## 3. Results

### 3.1. Transmission Electron Microscopy

**Figure 2:**
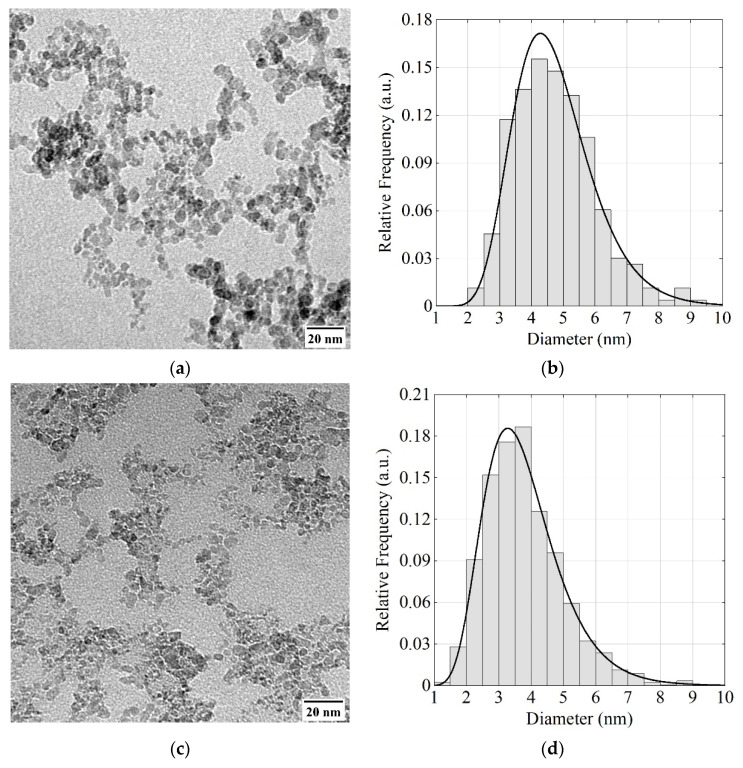
(**a**,**c**) Examples of TEM images of the DF-DND and S-DND samples; (**b**,**d**) size distributions for DF-DNDs and S-DNDs evaluated using all TEM images available.

Figure 2a,c show examples of TEM images of DF-DND and S-DND samples, respectively, obtained using FEI Tecnai G2 30 S-TWIN, NRC “Kurchatov Institute”—CRISM “Prometey”, Russia. For these measurements, a 2–3 mm^3^ sample of the powder was added to 1 mL of distilled water, and the container with the mixture was placed in an ultrasonic bath filled with water. It was then sonicated for 30 min. The resulting suspension (2–3 drops) was applied to a carbon replica placed on the plain grid manufactured by Pacific Grid-Tech. After drying, the replica was examined via TEM. 

The size distribution of DF-DNDs and S-DNDs shown in Figure 2b,d, respectively, was evaluated using all TEM images available, examples of which are shown in Figure 2a,b. For the presented histograms, we used 10 TEM images for S-DNDs and 1 TEM image for DF-DNDs. The visible projection of an individual particle was described by an ellipse. The particle was assigned a diameter equal to the diameter of a circle of equal area. The total number of particles in TEM images of S-DNDs was 2078, and the total number of DF-DND particles was 264.

The mean particle diameters in DF-DND and S-DND samples are ~4.7(2) nm and ~3.8(1) nm respectively. This difference in mean diameters is large enough for the purpose of this study, i.e., for a comparison of the two DND reflectors as a function of the particle size.

### 3.2. X-ray Diffraction

Another non-neutron method of evaluating DND sizes is the broadening of peaks in XRD patterns. 

The data were collected from DF-DND and S-DND powders by combining a powder diffractometer Rigaku SmartLab III (Rigaku Corporation, Tokyo, Japan) equipped with a CuKα source (λ=1.541 Å) and the ID28 diffractometer at ESRF (Grenoble, France) [47] with a PILATUS3 1M area detector (λ=0.784 Å). 

With the Rigaku diffractometer, a planar powder stage was used as the sample holder. With the ID28 diffractometer, the samples were packed in quartz capillaries with the diameter of 200 μm; the data were evaluated using SNBL Toolbox [48] and Dioptas [49] software.

**Figure 3:**
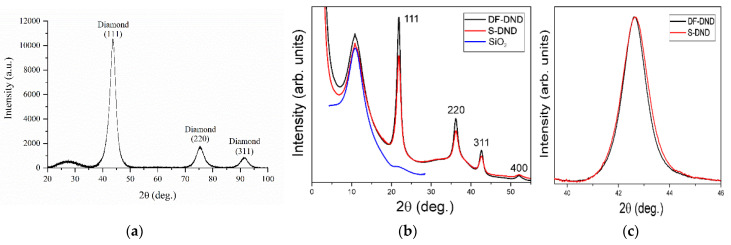
X-ray diffraction patterns: (**a**) S-DNDs at Rigaku SmartLab III; background is subtracted. (**b**) ESRF ID28 data for DF-DNDs and S-DNDs composed of three sets corresponding to three positions of the 2D detector; empty capillary scattering is shown. (**c**) Close-up of the 311 peak with the subtracted background and peak rescaling.

The results are shown in Figure 3. The positions of the diffraction peaks correspond to the diamond lattice. Broadening of the peaks contains information about particle sizes. A characteristic size of the coherent scattering region was determined by analyzing the full width of the peak at half maximum using the Sedyakov–Scherrer equation and the shape of the peak through the comparison of full width at one-fifth and four-fifths of the maximum [50]. For the latter purpose, the 311 peak was the most convenient, and its width change by ~10% is shown in Figure 3c with a parabolic local background subtracted and the peak height scaled. The results, as expected, are only slightly dependent on the procedure. Rigaku data provide the value of the mean particle size equal to ~3.4 nm for S-DND, while the mean size and dispersion of sizes from the synchrotron data are, respectively, ~4.1 nm and ~2.0 nm for DF-DND, and ~3.7 nm and 1.7 nm for S-DND. The mean value $D^{4}/D^{3}$ estimated from XRD peak broadening thus decreases by ~10%. 

### 3.3. Small-Angle Neutron Scattering

**Figure 4:**
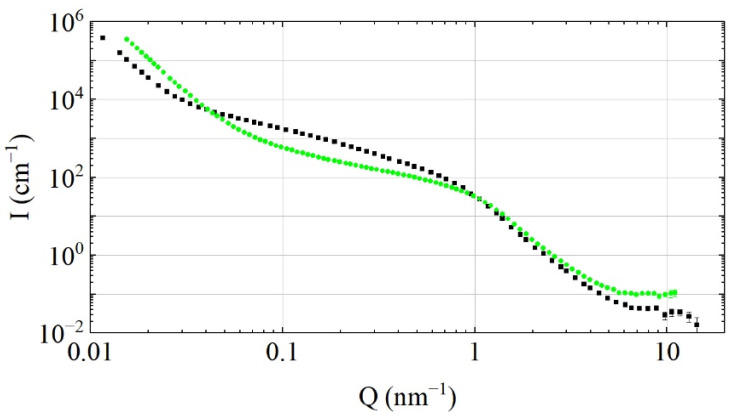
Intensity I (nm^−1^) of neutron scattering versus transferred momentum Q (nm^−1^) for DF-DNDs (black squares) and S-DNDs (green circles). To compare only the effect of particle sizes, and not powder density, both curves are normalized to the sample density of 1 g/cm^3^. SANS characterization was performed with YuMO, D11, and NGB30 instruments, and their neutron wavelengths and ranges of transferred momenta (Q) were equal to 0.7–5.0 Å and 7 × 10^−2^ nm^−1^ < Q < 10^1^ nm^−1^; 6 Å and 10^−2^ nm^−1^ < Q < 10^0^ nm^−1^; and 6 Å and 3.4 × 10^−2^ nm^−1^ < Q < 1.2 × 10^0^ nm^−1^, respectively. The Q-ranges used for the summary plot for the S-DNDs are as follows: 1.55 × 10^−2^ nm^−1^–8.53 × 10^−1^ nm^−1^ from D11 and 8.53 × 10^−1^ nm^−1^–1.10 × 10^0^ nm^−1^ from YuMO; for the DF-DNDs, they are as follows: 9.03 × 10^−3^ nm^−1^–1.55 × 10^−2^ nm^−1^ and 7.57 × 10^−2^ nm^−1^–9.85 × 10^−2^ nm^−1^ from NGB30, 1.55 × 10^−2^ nm^−1^–7.57 × 10^−2^ nm^−1^ from D11, and 9.85 × 10^−1^ nm^−1^–1.47 × 10^0^ nm^−1^ from YuMO.

SANS provides the most direct and unambitious information for the analysis of scattering and transport of slow neutrons in DND powders. SANS characterization [51] of DF-DND and S-DND samples was performed using three instruments: a time-of-flight spectrometer YuMO in the two-detector mode (FLNP, JINR, Dubna, Russia [52]), a diffractometer D11 (ILL, Grenoble, France) [53,54], and an NGB30 at the NIST Center for Neutron Research (Gaithersburg, MD, USA) [55], and their neutron wavelengths and ranges of transferred momenta (Q) were equal to 0.7–5.0 Å and 7 × 10^−2^ nm^−1^ < Q < 10^1^ nm^−1^; 6 Å and 10^−2^ nm^−1^ < Q < 10^0^ nm^−1^; and 6 Å and 3.4 × 10^−2^ nm^−1^ < Q < 1.2 × 10^0^ nm^−1^, respectively. The three SANS instruments were used to increase the reliability and precision of the results and to select Q-ranges with maximum statistical accuracy and minimum backgrounds. To match the SANS data measured at different Q-ranges (corresponding to different distances from the sample to the detector) as well as SANS data measured with the various instruments (YuMO, D11, and NGB30), the following procedures were applied: (1) With each instrument, a few Q-ranges were measured with a significant overlapping and high enough statistics in the overlapping ranges. The data from the overlapping Q-ranges were used to normalize the intensity for the entire Q-range for each instrument. (2) The absolute intensity of neutron scattering was normalized using the data measured with NGB30, where both the transmitted beam and the scattered intensity were recorded for each measurement. As the same DF-DNDs were used for all three instruments, we matched the absolute intensities taking into account the density of the DF-DND samples used with each instrument. As both DF-DND and S-DND samples were measured with two of the three instruments (D11 and YuMO), we could match the intensities of neutron scattering for the two samples. The Q-ranges used for the summary plot presented in Figure 4 for the S-DNDs are as follows: 1.55 × 10^−2^ nm^−1^–8.53 × 10^−1^ nm^−1^ from D11 and 8.53 × 10^−1^ nm^−1^–1.10 × 10^0^ nm^−1^ from YuMO; for the DF-DNDs, they are as follows: 9.03 × 10^−3^ nm^−1^–1.55 × 10^−2^ nm^−1^ and 7.57 × 10^−2^ nm^−1^–9.85 × 10^−2^ nm^−1^ from NGB30, 1.55 × 10^−2^ nm^−1^–7.57 × 10^−2^ nm^−1^ from D11, and 9.85 × 10^−1^ nm^−1^–1.47 × 10^0^ nm^−1^ from YuMO. The absolute calibration of scattering probabilities is an important input for the simulation of neutron propagation in powders. 

We used Igor macros [56] to evaluate the SANS data. The samples were placed inside aluminum 1 mm cells with 1 mm windows for measurements with YuMO and in Quartz SUPRASILL 1 mm cells with D11 and NGB30. The DF-DNDs bulk density was equal to 0.56 g/cm^3^. The bulk density of S-DNDs was equal to 0.67 g/cm^3^.

Figure 4 compares the merged results of SANS measurements made with all these instruments. The shape of the scattering intensities for the two samples differs significantly at small values Q < 10^0^ nm^−1^ due to the lower concentration of agglomerates in S-DNDs resulting from the DND size separation procedure. Another natural consequence of this procedure is that the number of individual DNDs (corresponding to large values of Q > 10^0^ nm^−1^) increases by 36%. These observations agree with our expectations based on the knowledge of the DND separation method. Incoherent scattering at Q > 7 × 10^0^ nm^−1^ is defined mainly by the presence of hydrogen. It is larger for S-DND powder because it is not fluorinated; however, this fact is not relevant to our calculations, as we do not consider the effect of impurities.

## 4. Discussion

### 4.1. Approximation of the Size Distribution of DNDs Using MDDNS

**Figure 5:**
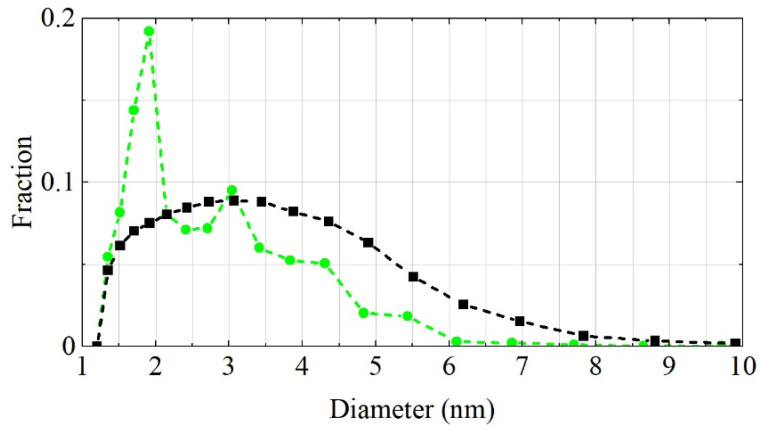
Size distribution of scatterers evaluated using MDDNS for S-DNDs (green circles) and DF-DNDs (black squares). Points correspond to the results of calculation.

As previously mentioned, our main goal was not to precisely extract the size distribution of real powders but to find an approximate model with a size distribution that allows us to precisely reproduce neutron scattering. Figure 5 presents the diameter distribution of ideal DNDs obtained from MDDNS in the case of DF-DND and S-DND samples evaluated using the SANS data shown in Figure 4 after the incoherent scattering on hydrogen in S-DNDs was subtracted. The physical basis for the procedure of size evaluation is the model of discrete-sized diamond nanospheres (MDDNS) [25,46], which represents the medium as ideal independent diamond nanospheres with a discrete set of diameters. Such a model medium scatters neutrons the same way as a real medium but also allows model extrapolation of the measured experimental data to other wavelength and angular ranges not accessible directly with standard SANS devices. The mathematical algorithm of this procedure will be described in detail in a forthcoming publication and is the subject of a patent (RU 2020662675).

Although both SANS curves in Figure 4 are of equal statistical quality, the size distribution for MDDNS DF-DNDs is smooth in shape, whereas that for S-DNDs shows considerable fluctuations. This difference might be because fewer nanodiamond diameters effectively contribute to the scattering of neutrons on S-DNDs. Such fluctuations have no effect on the precision of model calculations of neutron transport in S-DNDs on the condition that our model precisely describes the SANS data. In the range of 1.2–10.0 nm, the mean size of ideal scatterers in the S-DND model is ~2.9 nm, while it is ~3.8 nm for the DF-DND model.

Figure 5 confirms the conclusion drawn from the other methods of DND characterization; that is, in S-DNDs, the fraction of smaller particles is larger, while the larger particles disappear completely. Moreover, it can be clearly observed that the size distributions obtained from MDDNS using SANS data have a fraction of noticeably smaller particles than those obtained with other methods. The reason for this difference is that neutrons are not scattered only on individual DNDs but also on fluctuations in the density of the medium. They are sensitive to the entire structure of the powder. The scattering on pores between DNDs also contributes to this. In MDDNS, scattering on small pores, on non-diamond carbon, on specific types of agglomeration, on specific shapes of DNDs, etc. corresponds to scattering on small nanospheres. Note that to calculate the transport of neutrons in the powder, we only used SANS data. Other methods serve only for a better understanding of the peculiarities of powder modifications and the effect of these modifications. 

### 4.2. Albedo Calculations

To calculate the albedo for different cases, as discussed below, we used size distributions shown in Figure 5 in Section 3.3 obtained from SANS data for DF-DNDs and S-DNDs. First, we simulated the neutron albedo for a theoretical case of semi-infinite flat media; second we considered cases of final thickness (flat and spherical layers) with the same or different density. In the latter case, we used the densities of real samples. Note that when preparing samples for SANS, we aimed at minimum but stable density; for DNDs of different types, the density was notably different. For a powder of infinite thickness and a flat geometry, the albedo does not depend on density, and it is defined only by the absorption and scattering cross-sections. For a powder of a finite thickness, the fraction of neutrons passing through the layer depends on the powder density; thus, the albedo depends on density. For a spherical trap, the albedo also changes for the infinite powder thickness, since it depends on the ratio of the radius of curvature of the surface to the depth of the penetration of neutrons into the powder. The calculations were performed within MDDNS, using the Monte Carlo method [57] and our original software. Incident neutrons are isotropic. 

**Figure 6:**
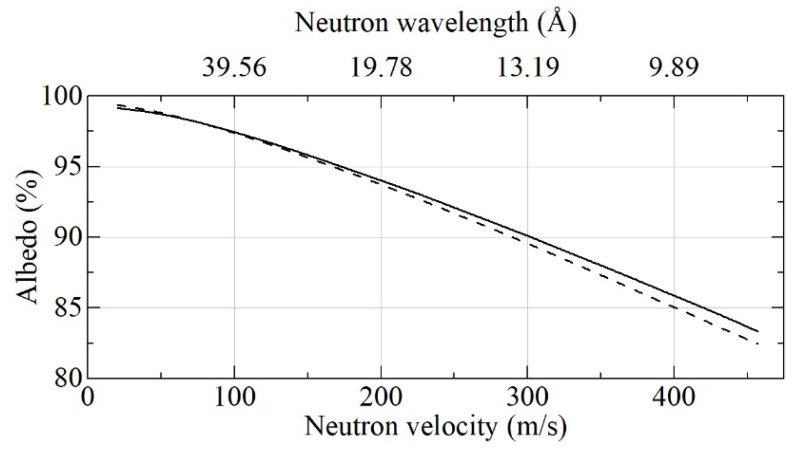
Neutron albedo for the flat semi-infinite media of DF-DNDs (dashed lines) and S-DNDs (solid lines) versus neutron velocity and wavelength.

Figure 6 shows the calculated neutron albedo from the semi-infinite media for DF-DND and S-DND powders. 

From Figure 6, it can be seen that for VCN velocities <70 m/s, the albedo is higher for DF-DNDs, whereas, for VCN velocities >70 m/s, it is higher for S-DNDs. This is explained by the presence of larger DNDs in DF-DNDs, which are closer to the optimal values for smaller velocities, and, vice versa, the presence of smaller DNDs in S-DNDs, which are closer to the optimal values for larger velocities. Both conclusions are in line with Formula (1) and the calculation presented in Figure 1. 

**Figure 7:**
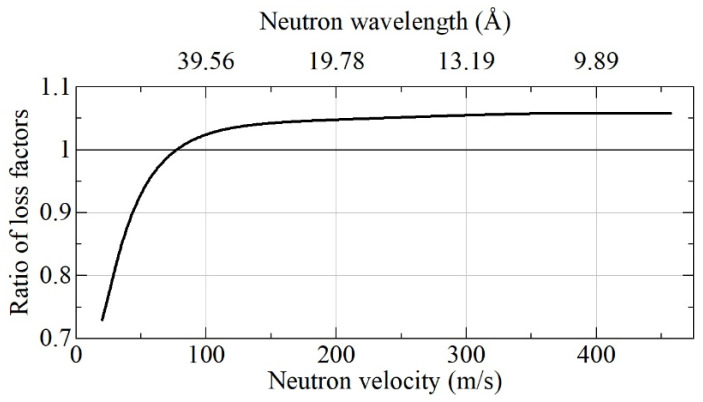
Ratio of loss factors η_(DF-DNDs)/η_(S-DNDs) at reflection from flat semi-infinite media of DF-DNDs and S-DNDs as a function of neutron velocity. The respective neutron albedo is shown in Figure 6.

The relatively small difference between these absolute albedo values is translated into a larger difference between loss factors (η = 1 – albedo), especially for albedo values close to 100%. As reciprocal loss factors are proportional to VCN densities, which can be accumulated in a trap with DND walls, we used the results shown in Figure 6 for the calculation of the loss factors ratio shown in Figure 7.

From Figure 7, it can be observed that DF-DNDs are more advantageous than S-DNDs for the reflection of neutrons with typical VCN velocities of ~50 m/s from semi-infinite flat media. However, when the goal is to increase the upper boundary of the VCN velocity range, S-DNDs are slightly more appropriate than DF-DNDs. It must be borne in mind, however, that the advantage of S-DND will become more evident for realistic 3D geometries of DND reflectors, which we consider below. 

**Figure 8:**
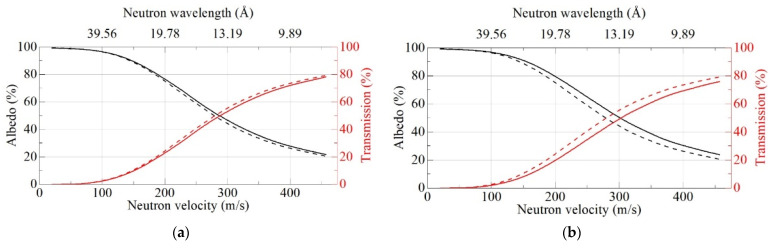
Neutron albedo (black lines) and transmission (red lines) for a flat layer of DF-DNDs (dashed lines) and S-DNDs (solid lines) versus neutron velocity [m/s]. (**a**) The layer thickness is 3 cm and the powder density is 0.56 g/cm^3^ for both samples; (**b**) the layer thickness is 3 cm and the powder density is 0.56 g/cm^3^ for DF-DNDs, and it is 0.67 g/cm^3^ for S-DNDs.

In contrast to the case of semi-infinite media, for a finite-thickness flat layer, powder density is important. Figure 8 shows the calculated neutron albedo from flat 3 cm-thick layers for DF-DNDs and S-DNDs for two cases: an equal density of the samples as well as for the real densities of SANS samples. This comparison allows one to separate the effects of powder density and particle sizes. The probability of neutron absorption is below 1.43% for S-DNDs, and it is below 1.30% for DF-DNDs, at any velocity. 

As Figure 8 clearly shows, for a finite thickness geometry, the albedo increases with an increase in powder density. This can be explained by the fact that increasing density is effectively equivalent to increasing thickness (for the same density). The effect of density is more pronounced in a 3D reflector geometry, which is of interest for a VCN source reflector or a VCN trap. It assumes that there is a cavity inside the DND reflector. This effect can be explained by the fact that the probability of return of a VCN to the cavity decreases with an increase in the depth of penetration into the reflector.

**Figure 9:**
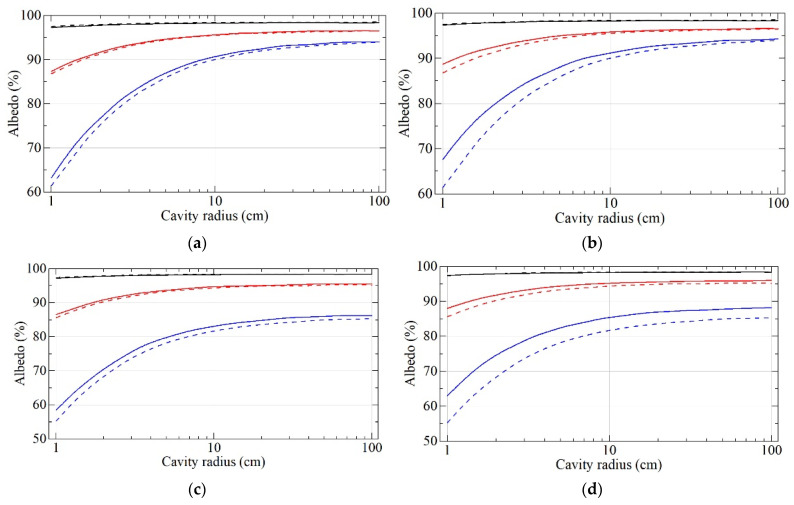
Neutron albedo for the velocities of 50 m/s (black lines), 100 m/s (red lines), and 150 m/s (blue lines) for S-DNDs (solid lines) and DF-DNDs (dashed lines) versus the cavity radius. (**a**) A powder layer of infinite thickness; the density is 0.56 g/cm^3^ for both samples. (**b**) A powder layer of infinite thickness; the density is 0.56 g/cm^3^ for DF-DNDs, and it is 0.67 g/cm^3^ for S-DNDs. (**c**) A powder layer with the thickness of 3 cm; the density is 0.56 g/cm^3^ for both samples. (**d**) A powder layer with the thickness of 3 cm; the density is 0.56 g/cm^3^ for DF-DNDs, and it is 0.67 g/cm^3^ for S-DNDs.

Figure 9 shows the results of calculations of the neutron albedo from the wall of a spherical cavity depending on the cavity radius. Neutron velocities are 50 m/s, 100 m/s, and 150 m/s; powder thickness is infinite and 3 cm; and powder densities vary.

In contrast to the flat geometry, in a 3D geometry, the effect of density is significant provided the cavity radius is comparable with the depth of penetration of VCNs into the reflector. As shown in Figure 9, this condition is always met for any cavity radius and powder type when the goal is to increase the upper boundary of the velocity range of efficient reflection of VCNs. In this case, VCNs with a maximum velocity from the range penetrate into the DND wall to a depth comparable to the cavity radius. 

One could try to compress powders to a higher density. However, there are certain constraints. With the decrease in the DND density, at some point, the density fluctuations would also decrease, thus resulting in a decrease in neutron scattering. The powder becomes more transparent to neutrons, and as it approaches the diamond density, it becomes completely transparent. This phenomenon can develop unevenly. In some areas of the compressed powder, the density may become too high, and in others, it may not. A technical realization of this compactification and the compressibility of DNDs of different types are the topic of a separate study.

**Figure 10:**
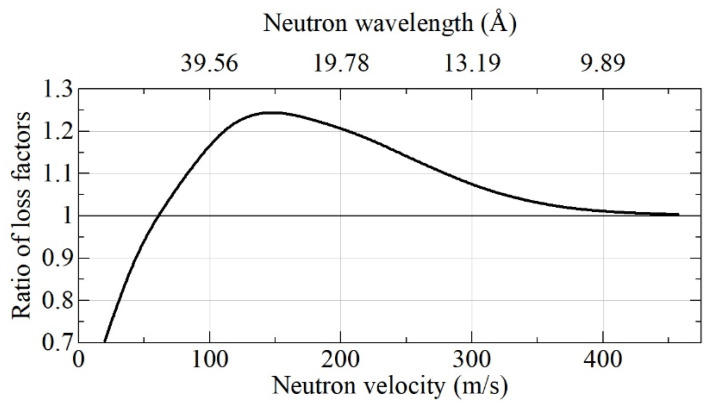
Ratio of loss factors $\eta_{\mathrm{DF}-\mathrm{DNDs}}/\eta_{S-\mathrm{DNDs}}$ at reflection from the wall of a spherical cavity with a radius of 5 cm and a wall thickness of 3 cm for realistic powder densities of 0.56 g/cm^3^ for DF-DNDs and 0.67 g/cm^3^ for S-DNDs.

To summarize the results of the present study, we compare the performance of reflectors based on DF-DNDs and S-DNDs for a realistic geometry of a spherical cavity with a radius of 5 cm and a wall thickness of 3 cm for realistic powder densities (see Figure 10).

A comparison of Figure 7 and Figure 10 shows that (1) S-DNDs are more advantageous for the reflection of VCNs starting from a smaller velocity equal to ~60 m/s; (2) the decrease in the loss factor for S-DNDs is also more important, and it is equal to ~20–25% in a broad range of VCN velocities. The first point is important when estimating the spectrum of VCNs to be reflected with a DND reflector. The second factor is directly translated into the gain in the intensity of VCNs, which can be accumulated in DND traps with S-DND and DF-DND walls, respectively. The relative decrease in the gain factor in Figure 10 is a result of the partial transmission of faster neutrons through the wall of a finite thickness. 

As Formula (1) shows, the optimum diameter of DNDs for VCNs with a velocity of ~50 m/s is ~4.3 nm. If the goal is to increase the upper boundary of the velocity range of effective reflection of VCNs, the mean diameter of DNDs must be reduced. However, it cannot be reduced by more than a factor of approximately three because even smaller DNDs do not exist, and because the cross-section of coherent scattering of neutrons decreases dramatically as a function of the DND diameter (as the sixth power of the diameter [7]). If the goal is to improve storage times for a softer part of the VCN spectrum, the mean diameter of DNDs must be increased. However, it should not be increased by more than a factor of approximately three, because there are other mechanisms of reflection of slower VCNs that are efficient for such velocities (optical Fermi potential and supermirrors). These arguments roughly define the range of effective reflection of VCNs by DNDs and guide the choice of parameters of DNDs for the design of DND reflectors optimized for the diffusive reflection of VCNs. 

## 5. Conclusions

In the current study, we investigated the effect of particle sizes on the efficiency of DND neutron reflectors. If typical DNDs with a size of ~4.3 nm are efficient for the reflection of typical VCNs with a velocity of ~50 m/s, other mean sizes are more efficient for other VCN velocities. Thus, up to a factor of approximately three, reduction in the mean size allows more efficient reflection of faster neutrons. An increase up to a factor of approximately three in the mean size allows the storage times of slower VCNs to be increased. Simulations based on SANS data and MDDNS (model of discrete-size diamond nanospheres) for a realistic reflector geometry show that the particle size reduction corresponding to the replacement of DF-DNDs (DNDs with the best achieved performance) by S-DNDs (DNDs of smaller sizes) increases neutron albedo in the broad velocity range above ~60 m/s. This increase in albedo results in an increase in the density of faster VCNs in such a reflector cavity of up to ~25%, as well as an increase in the upper boundary of velocities of efficient VCN reflection. As the density of DND reflectors is of importance for their efficiency, an investigation of the practical feasibility of its increase and corresponding feedback is of interest for future research. 

## 6. Patents

The algorithm for extracting a model-independent size distribution of scatterers from small-angle scattering data was developed to obtain the results reported in this manuscript. It is protected by the author’s certificate of state registration of the software “Structural Nanopowders Analyzer Based on Small-Angle Scattering Data (SNASAS)” RU 2020662675, issued by the Federal Service for Intellectual Property. 

## Data Availability

Neutron data were obtained in experiments at ILL, Grenoble, France: doi:10.5291/ILL-DATA.3-07-386; doi:10.5291/ILL-DATA.3-07-361.
